# Supplementary material for: Risk Reclassification of Patients with Endometrial Cancer Based on Tumor Molecular Profiling: First Real World Data
Source: J Pers Med. 2021 Jan 15;11(1):48. doi: 10.3390/jpm11010048 (PMC7830511; doi:10.3390/jpm11010048)
Supplement: Supplementary file 1 [file jpm-11-00048-s001.zip › Supplemental Figures_final.docx]

S1 Supplemental figures

Figure 1


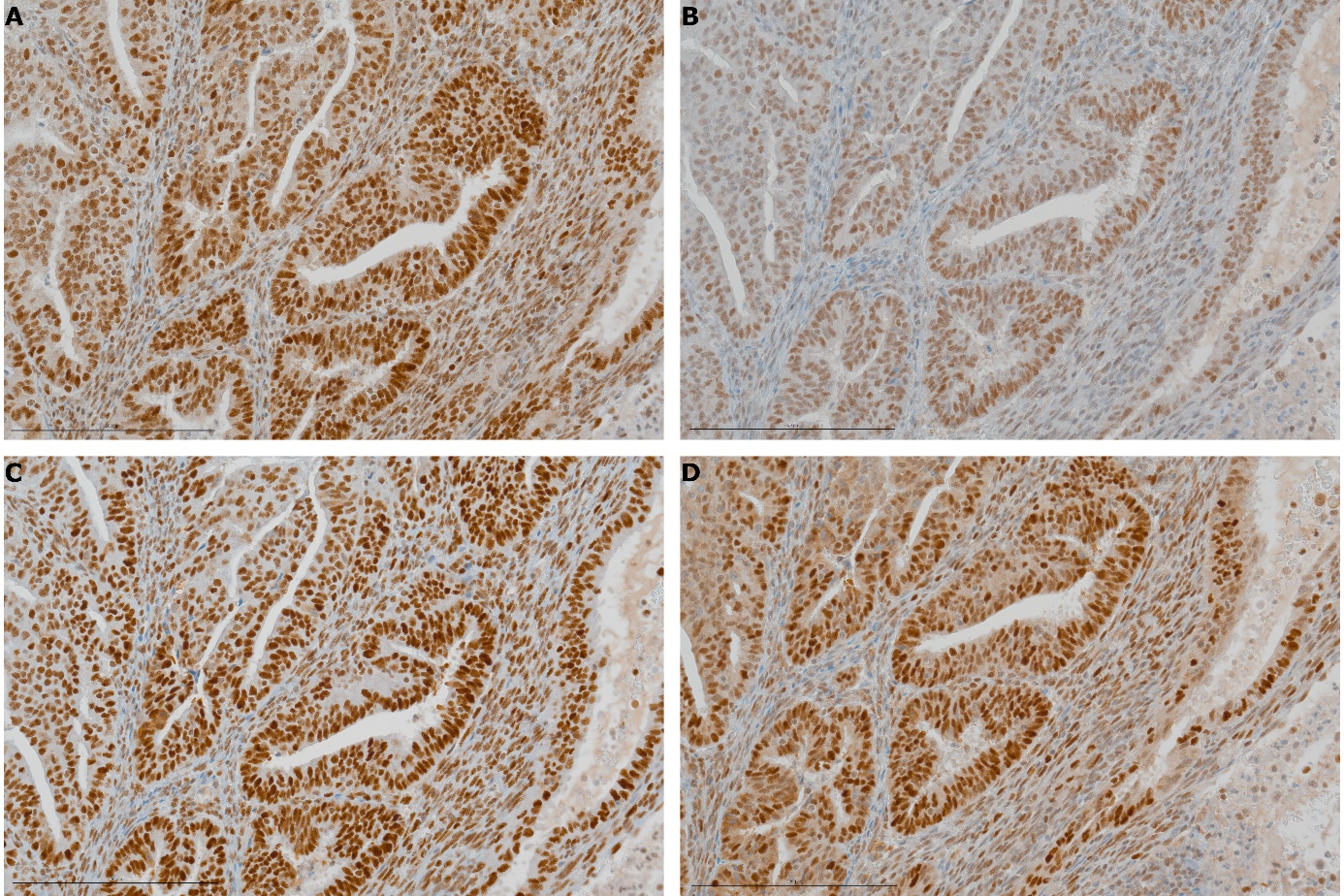


Representative immunohistochemical photomicrography of MMR proficient endometrial cancer: MLH1 (A), PMS2 (B), MSH2 (C), MSH6 (D). Cancer cells show retained nuclear immunoreactivity. Normal (stromal) cells serve as internal postitive control. (Magnification 20x for all images, scale bar = 200µm).

Figure 2


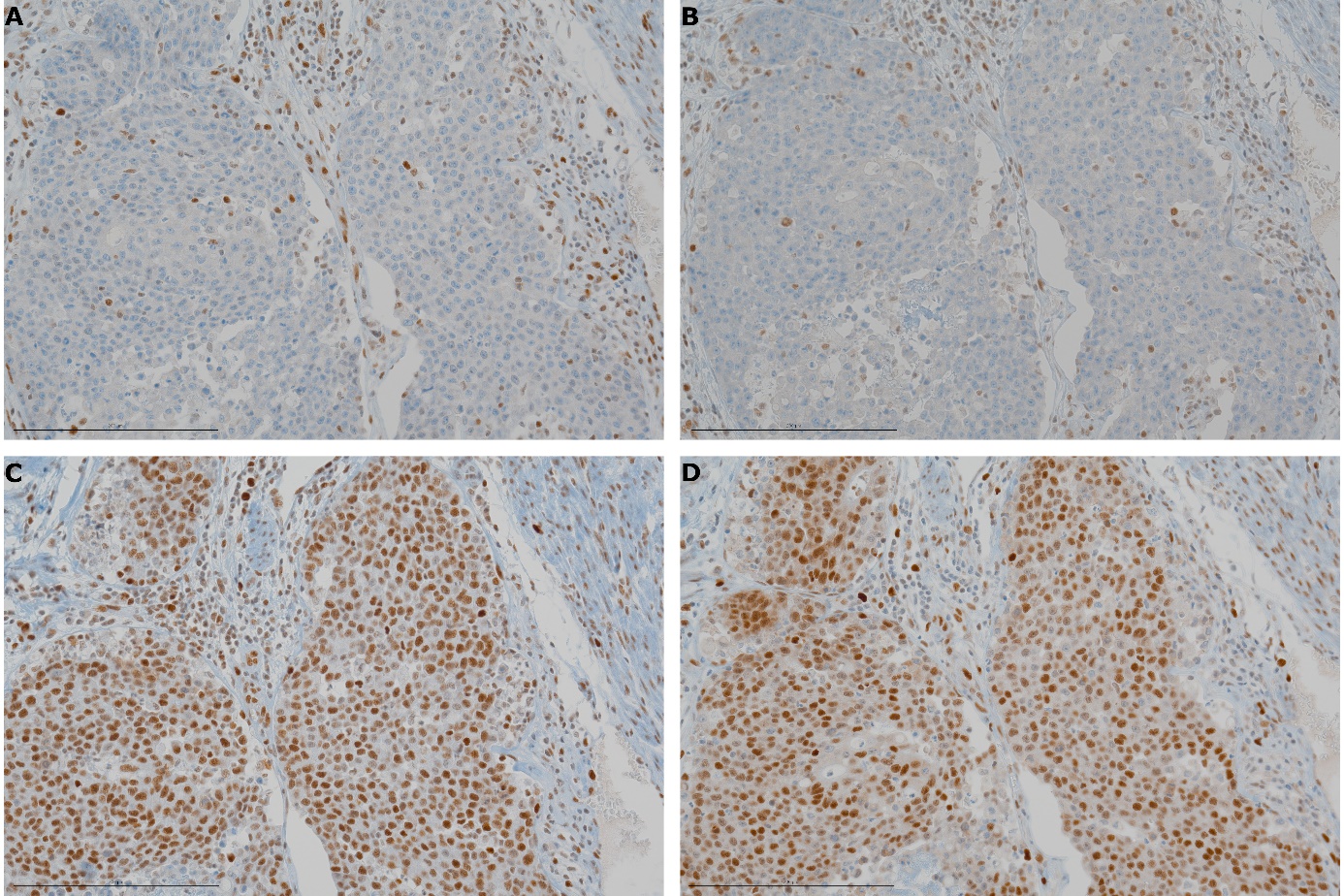


Representative immunohistochemical photomicrography of MMR deficiency in endometrial cancer: MLH1 (A), PMS2 (B), MSH2 (C), MSH6 (D). Loss of MLH1 and PMS2 protein expression in all carcinoma cells, while MSH2 and MSH6 shows retained nuclear immunoreactivity. Normal (stromal) cells serve as internal positive control. (Magnification 20x for all images, scale bar = 200µm).

Figure 3


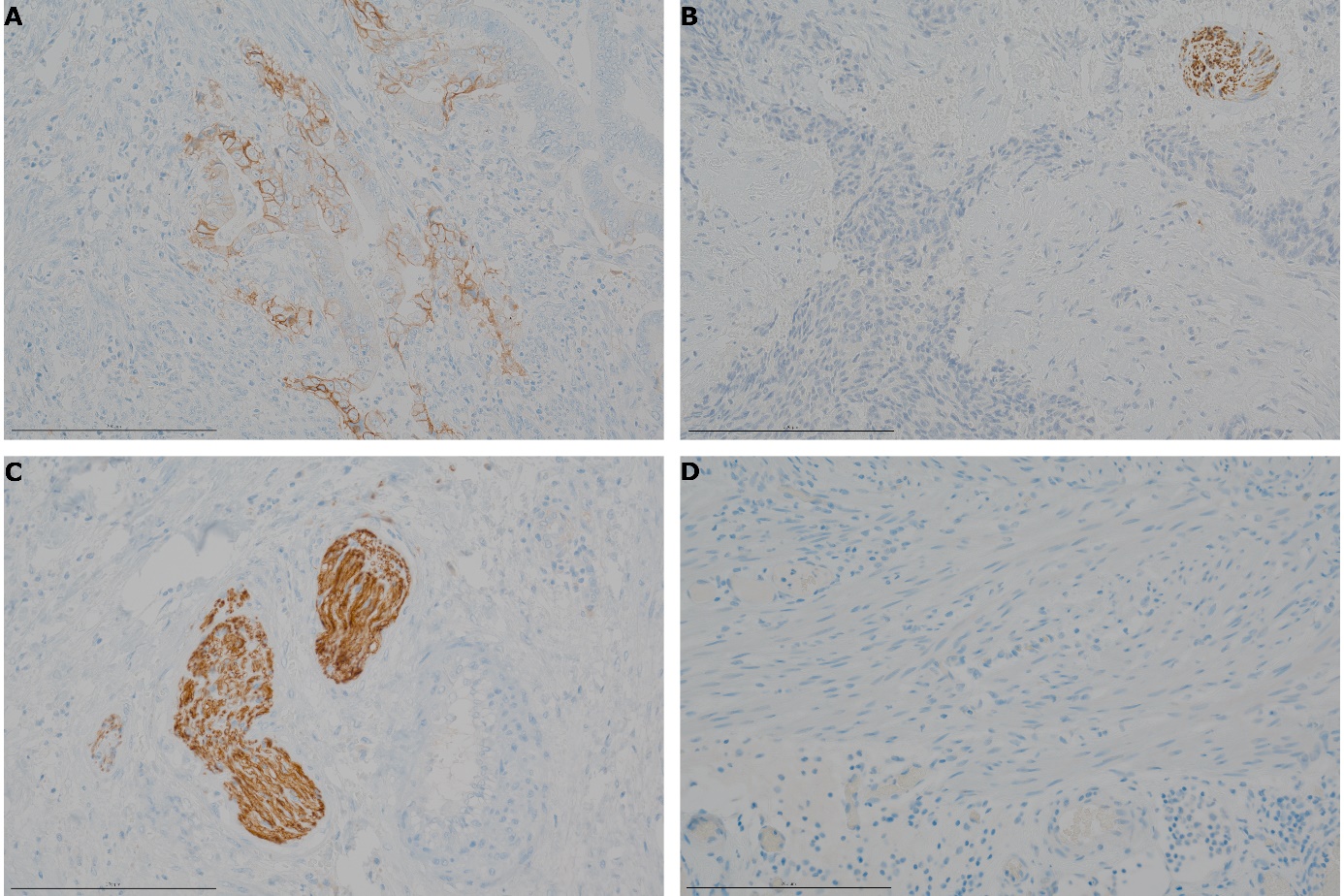


Representative immunohistochemical photomicrography of L1CAM and controls. L1CAM expression in cancer cells (A), no immunoreactivity for L1CAM in cancer cells and positive immunostaining of a peripheral nerve, serving as internal positive control (B). L1CAM control slide with a peripheral nerve to ensure accurate staining (C) and isotype-specific antibody (Negative Control (Monoclonal) – Ventana) to preclude unspecific binding (D). (Magnification 20x for all images, scale bar = 200µm).
